# OpenSim: Simulating musculoskeletal dynamics and neuromuscular control to study human and animal movement

**DOI:** 10.1371/journal.pcbi.1006223

**Published:** 2018-07-26

**Authors:** Ajay Seth, Jennifer L. Hicks, Thomas K. Uchida, Ayman Habib, Christopher L. Dembia, James J. Dunne, Carmichael F. Ong, Matthew S. DeMers, Apoorva Rajagopal, Matthew Millard, Samuel R. Hamner, Edith M. Arnold, Jennifer R. Yong, Shrinidhi K. Lakshmikanth, Michael A. Sherman, Joy P. Ku, Scott L. Delp

**Affiliations:** 1 Department of Bioengineering, Stanford University, Stanford, California, United States of America; 2 Department of Mechanical Engineering, Stanford University, Stanford, California, United States of America; 3 Department of Orthopaedic Surgery, Stanford University, Stanford, California, United States of America; Hebrew University of Jerusalem, ISRAEL

## Abstract

Movement is fundamental to human and animal life, emerging through interaction of complex neural, muscular, and skeletal systems. Study of movement draws from and contributes to diverse fields, including biology, neuroscience, mechanics, and robotics. OpenSim unites methods from these fields to create fast and accurate simulations of movement, enabling two fundamental tasks. First, the software can calculate variables that are difficult to measure experimentally, such as the forces generated by muscles and the stretch and recoil of tendons during movement. Second, OpenSim can predict novel movements from models of motor control, such as kinematic adaptations of human gait during loaded or inclined walking. Changes in musculoskeletal dynamics following surgery or due to human–device interaction can also be simulated; these simulations have played a vital role in several applications, including the design of implantable mechanical devices to improve human grasping in individuals with paralysis. OpenSim is an extensible and user-friendly software package built on decades of knowledge about computational modeling and simulation of biomechanical systems. OpenSim’s design enables computational scientists to create new state-of-the-art software tools and empowers others to use these tools in research and clinical applications. OpenSim supports a large and growing community of biomechanics and rehabilitation researchers, facilitating exchange of models and simulations for reproducing and extending discoveries. Examples, tutorials, documentation, and an active user forum support this community. The OpenSim software is covered by the Apache License 2.0, which permits its use for any purpose including both nonprofit and commercial applications. The source code is freely and anonymously accessible on GitHub, where the community is welcomed to make contributions. Platform-specific installers of OpenSim include a GUI and are available on simtk.org.

This is a *PLoS Computational Biology* Software paper.

## Introduction

**Fig 1:**
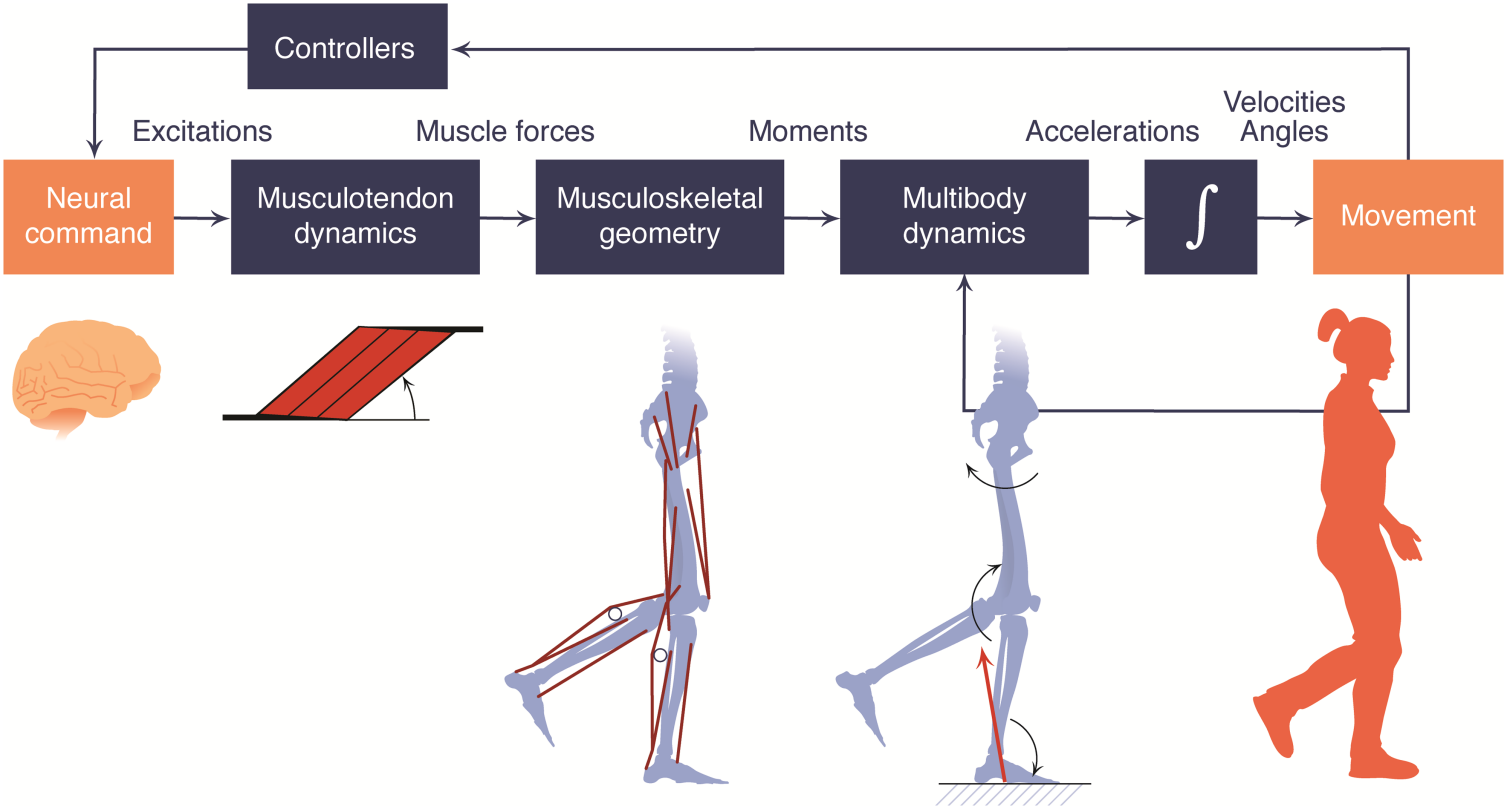
Elements of a typical musculoskeletal simulation in OpenSim. Movement arises from a complex orchestration of the neural, muscular, skeletal, and sensory systems. OpenSim includes computational models of these systems, enabling prediction and analysis of human and animal movement. Neural command to muscles, in the form of excitations, can be estimated from controller models or experimental data (e.g., EMG). OpenSim’s Hill-type musculotendon models, which translate excitations into muscle forces, include the force–length and force–velocity properties of muscles. OpenSim provides the flexibility to represent the wide range of muscle geometry found in animals, and the parameters defining muscle geometry and contraction dynamics can be modified based on experimental data. OpenSim’s underlying Simbody engine for multibody dynamics includes contact models (e.g., to simulate foot–ground interaction) and several solvers/integrators that allow users to generate muscle-driven simulations (forward simulation) or to solve for muscle forces and moments that generate an observed motion (inverse simulation).

By studying the biomechanical structures and neuromuscular control underlying movement, we can discover strategies to prevent injury, treat disease, and enhance performance. The benefits are undeniable: an understanding of typical and impaired neuromuscular control has improved rehabilitation for patients after a stroke [1]; musculoskeletal analysis has shown promise as a tool for predicting outcomes of orthopaedic surgeries and for optimizing assistive devices [2, 3]; studies of posture have led to recommendations for establishing safe working conditions to reduce the risk of musculoskeletal injuries, such as carpal tunnel syndrome and low back pain [4, 5]; and biomechanical observations have led to improved technique and performance in swimming [6, 7]. However, the complex interactions of neural control with musculoskeletal dynamics during the production of movement (Fig 1) pose a significant barrier to making such discoveries. A complication is that many quantities of interest—including neural control signals and joint loads—are difficult or impossible to measure with experiments. To advance movement science, researchers desire computational modeling and simulation tools that span disciplines such as anatomy, physiology, neuroscience, kinesiology, mechanics, robotics, and computer science, distributed with permissive software licenses so that discoveries can be widely shared. Several open-source software packages (e.g., BTK [8] and OpenMA [9]) provide tools for collecting and analyzing experimental movement data, but have limited support for simulation and optimization tasks. While a few groups around the world have developed their own simulation and optimization software tools, these independent approaches limit the exchange of models and algorithms.

OpenSim enables the advancement of movement science by equipping research and clinical communities with biomechanical models and simulation tools in an open-source, extensible, and collaborative platform. OpenSim’s capabilities span four core areas. First, users can **build, manipulate, and interrogate** biomechanical models. For example, bone specimens were used to build a musculoskeletal model of the *Australopithecus afarensis* hand to investigate whether this primate species had sufficient grip strength to make certain stone tools [10]. Second, OpenSim can be used to **simulate** musculoskeletal dynamics and neuromuscular control. Simulations enable researchers to pursue studies that are difficult to perform experimentally, such as investigating how humans and animals exploit tendon elasticity to make running more efficient [11–13] and optimizing the design of implantable mechanisms and assistive devices [14–18]. Third, using solely principles of neuromuscular control and dynamic simulation, OpenSim can be used to **predict** novel movements and adaptations to novel conditions, without performing any experiments. This capability has led to a deeper understanding of muscle coordination during loaded and inclined walking [19], insight into limitations of reflexes in preventing ankle injuries when landing [20], and suggestions of optimal device design to enhance jumping performance [21]. Fourth, OpenSim’s modular and extensible design allows researchers to **create and share** new computational models [22, 23], numerical methods, and simulation tools [24–26] that extend the capabilities of the software.

**Fig 2:**
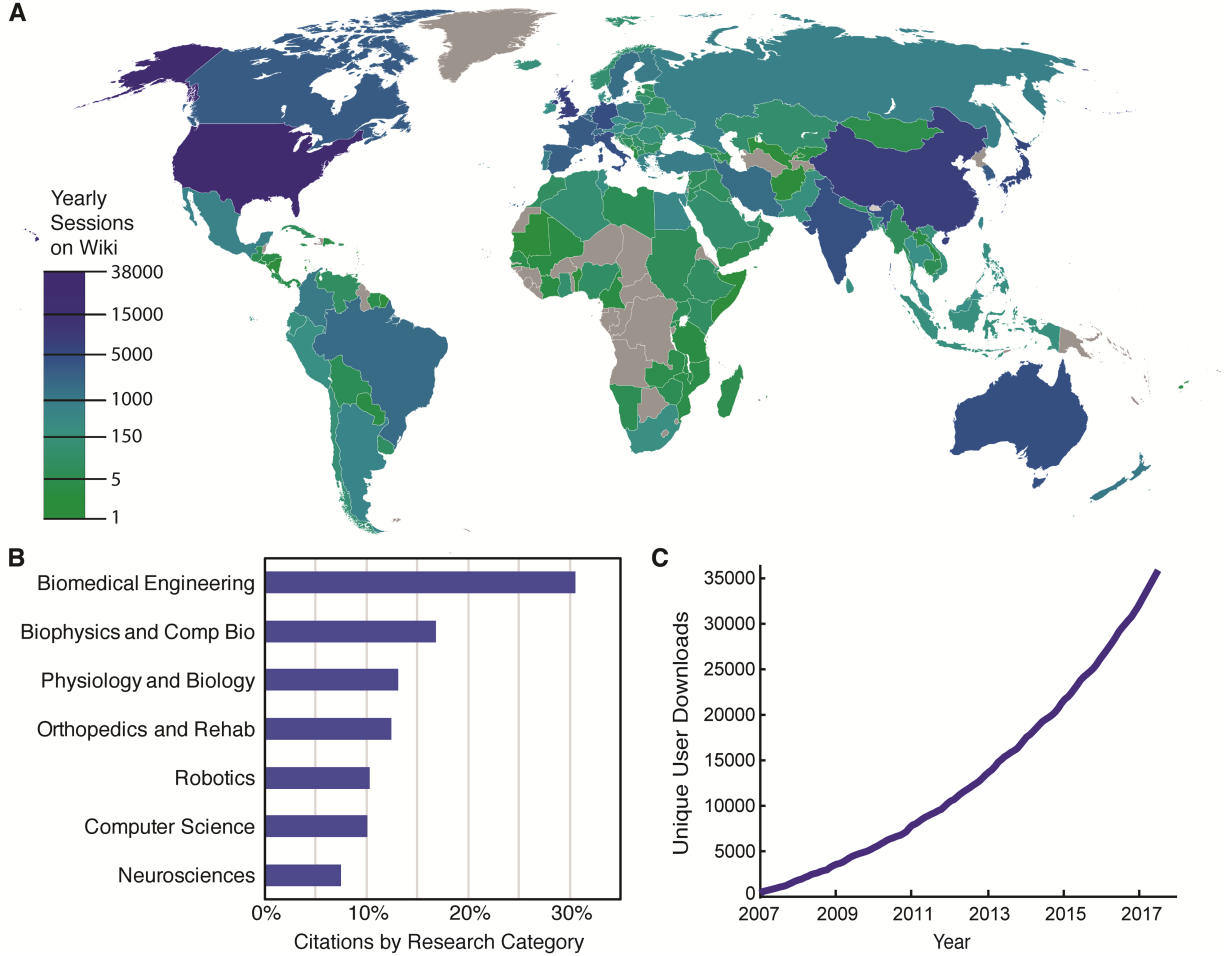
The OpenSim community is worldwide, diverse, and growing. (A) Locations of visitors to the OpenSim documentation (sessions per country in the 1-year period ending April 21, 2018). Since its launch in 2012, the OpenSim documentation wiki has been visited by over 25,000 users from around the world per year [30]. (B) Publications citing OpenSim by research category (Web of Science). Note that journals, and thus citations of OpenSim, may belong to more than one research category. According to Google Scholar, OpenSim [27] has been cited 1947 times as of June 13, 2018; based on analysis of the subset of these papers published in 2016, we estimate that 3/4 of these publications make use of the software. (C) Cumulative downloads of OpenSim since its release in August 2007. 35,915 users have downloaded the software as of June 13, 2018 [31]. World map in (A) created using tools at http://gunn.co.nz/map.

In addition to its advanced computational tools, OpenSim provides a collaborative research platform that serves a diverse, global, and expanding user base (Fig 2). This active research community is using OpenSim to make scientific discoveries, and is disseminating these results in workshops, webinars, conference presentations, and journal publications. The paper describing the first version of OpenSim [27] was cited 322 times in 2016 alone (Google Scholar; accessed June 5, 2017); approximately 3/4 of the citing papers used the software in their study. Many of the models, data, and plugins described in these references are publicly shared, enabling one to reproduce, validate, and extend others’ results. There are currently over 180 OpenSim-related projects on simtk.org, a website for sharing biophysical models and software, many of which contain valuable experimental data sets (e.g., [22, 28, 29]). OpenSim users also contribute to the community by asking and answering questions on the user forum and by contributing software to the codebase on GitHub. Community engagement is critical as user requirements play a substantial role in shaping the software. We have developed a suite of teaching materials, which includes (i) a user’s guide, (ii) dozens of tutorials and examples for training novice users, demonstrating advanced features, and providing templates for starting new studies, and (iii) documentation of the Application Programming Interface (API) for plugin developers and scripting users. The software and these teaching materials are used for introducing biomechanics to a broad range of students, from middle-school to graduate-school levels.

**Fig 3:**
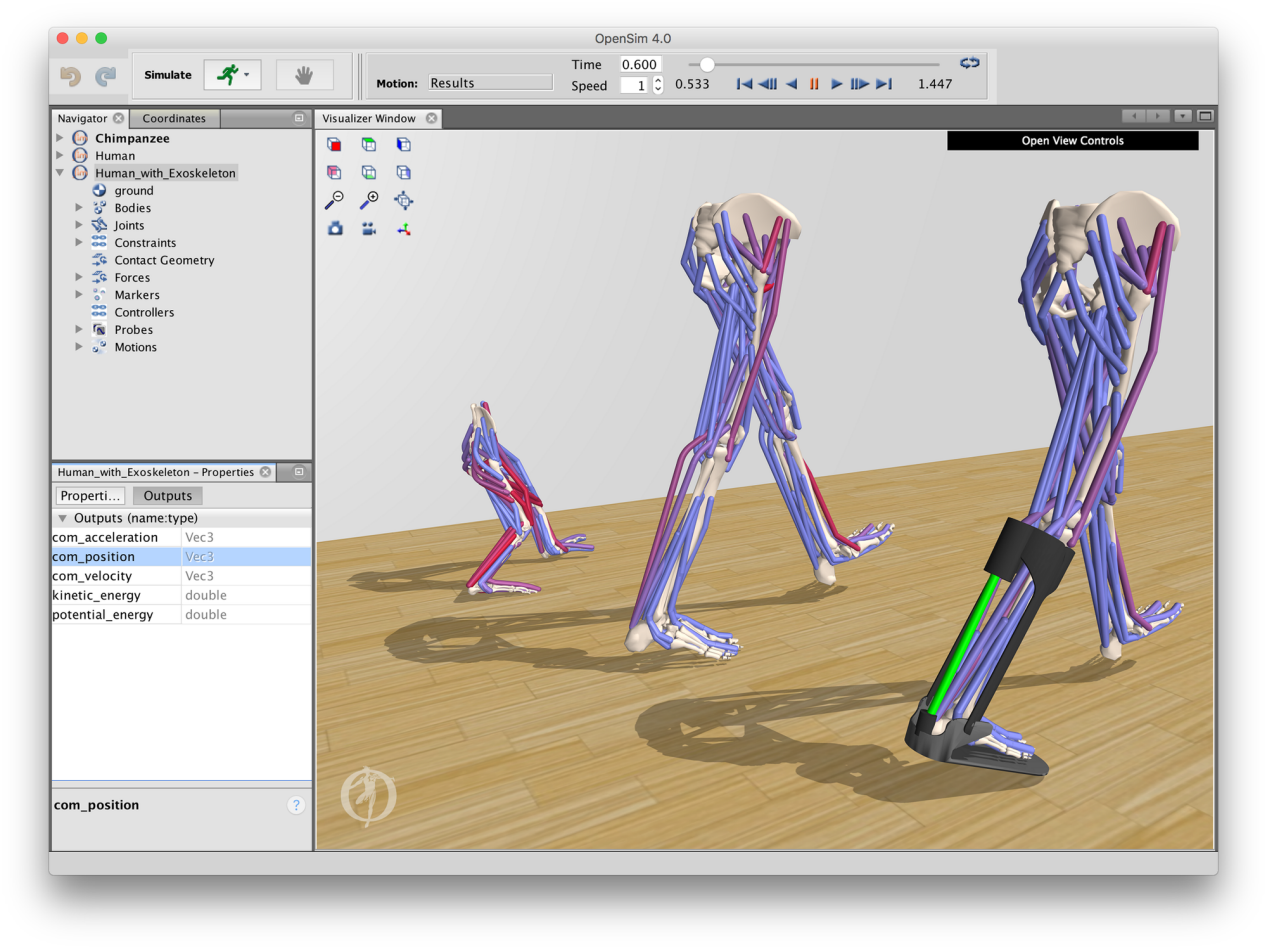
The OpenSim desktop application. A graphical user interface provides access to tools for inspecting, modifying, and simulating musculoskeletal models. Shown here are the results of muscle-driven simulations of human and chimpanzee walking that were generated by tracking experimental motion capture data. OpenSim models can be augmented with passive and active devices to explore designs of exoskeletons. (Human model and simulation from Rajagopal et al. [34]; chimpanzee model from O’Neill et al. [35] and unpublished simulation results provided by M.C. O’Neill and B.R. Umberger.)

This paper describes the design and capabilities of OpenSim and provides a sampling of the types of research questions that can be answered using OpenSim. The software was first released in August 2007 as a research tool for generating simulations of movement. Early versions of the software were used primarily for studying human gait and exploring the effects of pathologies and treatments [27, 32]. We have introduced many additional capabilities in recent years, among which are enhancements in four key areas. First, new **accurate models** for modeling muscle contraction dynamics, muscle metabolic power, joint kinematics, and assistive devices have been introduced. These models enable users to closely replicate human and animal movements, and to explore metabolic cost under natural and engineered conditions. An extensive test suite ensures these models are correctly implemented, robust, and efficient. Second, users are now able to explore beyond predefined workflows and create **custom studies** that combine existing computational tools in new ways. For example, users can now access muscle-related variables like moment arms [33], fiber lengths, and passive fiber forces directly from a model, without running a complicated analysis in the OpenSim application. This functionality is available through the C++ API as well as MATLAB and Python scripting interfaces, ensuring the tools are accessible to researchers with differing computational backgrounds. Third, OpenSim now includes functionality for controlling **data flow**, such as converting data from motion capture file formats and managing data within a simulation. Without the help of third-party tools, users can read in experimental data from files in standard motion capture file formats to plot the data in OpenSim and use it to generate simulations. Fourth, the OpenSim desktop application incorporates a modern **cross-platform visualizer** with tools for creating animations of movement (Fig 3), which provides users with more control over the visualization and allows use on both Windows and Mac operating systems. These capabilities enable groundbreaking work in the academic, clinical, and industrial arenas.

## Design and implementation

### Overview of the software design

**Fig 4:**
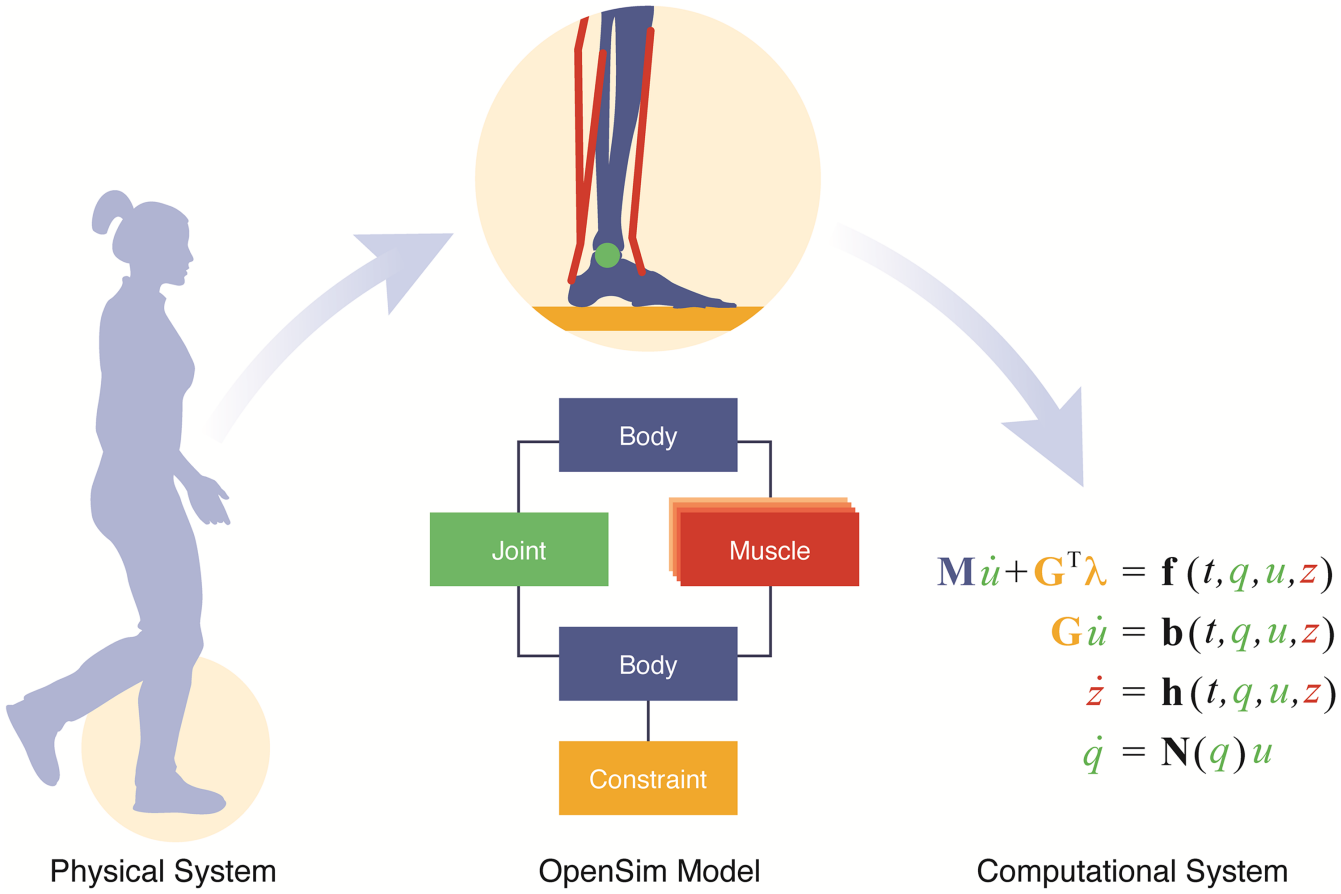
The OpenSim framework is used to study the dynamics of human and animal musculoskeletal systems. An OpenSim Model is a codified description of a physical system and its dynamics, and can be expressed as a topological graph of interconnected components. Each component represents a self-contained module (biological structure, neuromotor controller, mechatronic device, etc.) comprising the Model, and contributes to building the computational system. The computational system consists of two parts: (1) the system of equations (“System”), which includes physical parameters that are constant during a simulation (mass, dimensions, muscle properties, etc.); and (2) the State, which is the list of all variables in the System that may vary over time (e.g., joint angles). The model developer designs an OpenSim Model that represents the physical system of interest, and the OpenSim software automatically constructs the computational system of differential and algebraic equations that describe the dynamics of the Model.

Formulating and solving the equations that govern the motion of a neuromusculoskeletal system are daunting, even for experts. OpenSim automates the difficult and error-prone task of formulating these equations from a conceptual model and provides tools to solve them (Fig 4). We define a Model to be a codified description of the form (topology) and function (dynamics) of a biomechanical system, which can include neural, muscular, and skeletal structures, as well as non-biological components like exoskeletons. We capitalize “Model” here, and similar terms elsewhere, to denote the specific data structure (i.e., class) defined in OpenSim. An OpenSim user creates a Model by defining the components of the Model (e.g., rigid bodies and muscles), their properties (e.g., body masses and muscle fiber lengths), and the connections between each of the components (e.g., the femur and tibia bodies in a lower limb model are connected by a knee joint). OpenSim then automatically generates a System, which comprises the system of equations that governs the kinematics and dynamics of a Model. The System, which does not change during a simulation, is separate from the time-varying State of a Model, which stores the values of the variables in the Model’s equations of motion (e.g., joint angles, muscle activations, and the stretch of a clutched elastic cord). A simulation generates a trajectory of these States in time. One can then employ OpenSim’s Solvers to systematically study Models and their motion. For example, one can solve for a set of muscle forces required to track a movement observed experimentally, or integrate the Model’s equations of motion to compute its trajectory (States) over time.

OpenSim is accessible via a desktop application and the API, through C++, Java, MATLAB, and Python interfaces. The desktop application (built using the Java interface) comprises a graphical user interface (GUI) and a visualizer, enabling a broad community of users to apply models and simulations to study movement. The desktop application provides tools for users to visualize Models and motions via WebGL, interrogate and edit Models, configure and run Solvers, and plot and export simulation results. OpenSim’s command-line executables allow batch processing. Extensible Markup Language (XML) files are used to document and store Models and simulation parameters, and human-readable file formats allow users to archive and share simulation results. OpenSim is built atop other validated packages. OpenSim relies on Simbody to compute the dynamics of multibody systems, which is done using an order-*N* recursive formulation [36–39]. Additional dependencies include BTK (Coordinate 3D (C3D) file support), SWIG (creating non-C++ interfaces), doxygen (API documentation), Java NetBeans (GUI), JFreeChart (plotting), Jython (GUI scripting), and TinyXML (XML parsing).

OpenSim employs several design, implementation, and testing strategies to maintain software quality. The software’s object-oriented design strictly limits the scope of each software unit, allowing each to be tested thoroughly in isolation. We also use comprehensive unit testing to ensure components are valid (e.g., tests for Components, like Joints, verify that energy is conserved) and can be written to and read from file to maintain backward compatibility with old model files (serialization). Regression testing ensures that simulation and analysis results are preserved as the software grows and evolves. We employ continuous integration (via AppVeyor and Travis CI) to run our full test suite before accepting proposed changes to the codebase.

### Biologically accurate models

**Fig 5:**
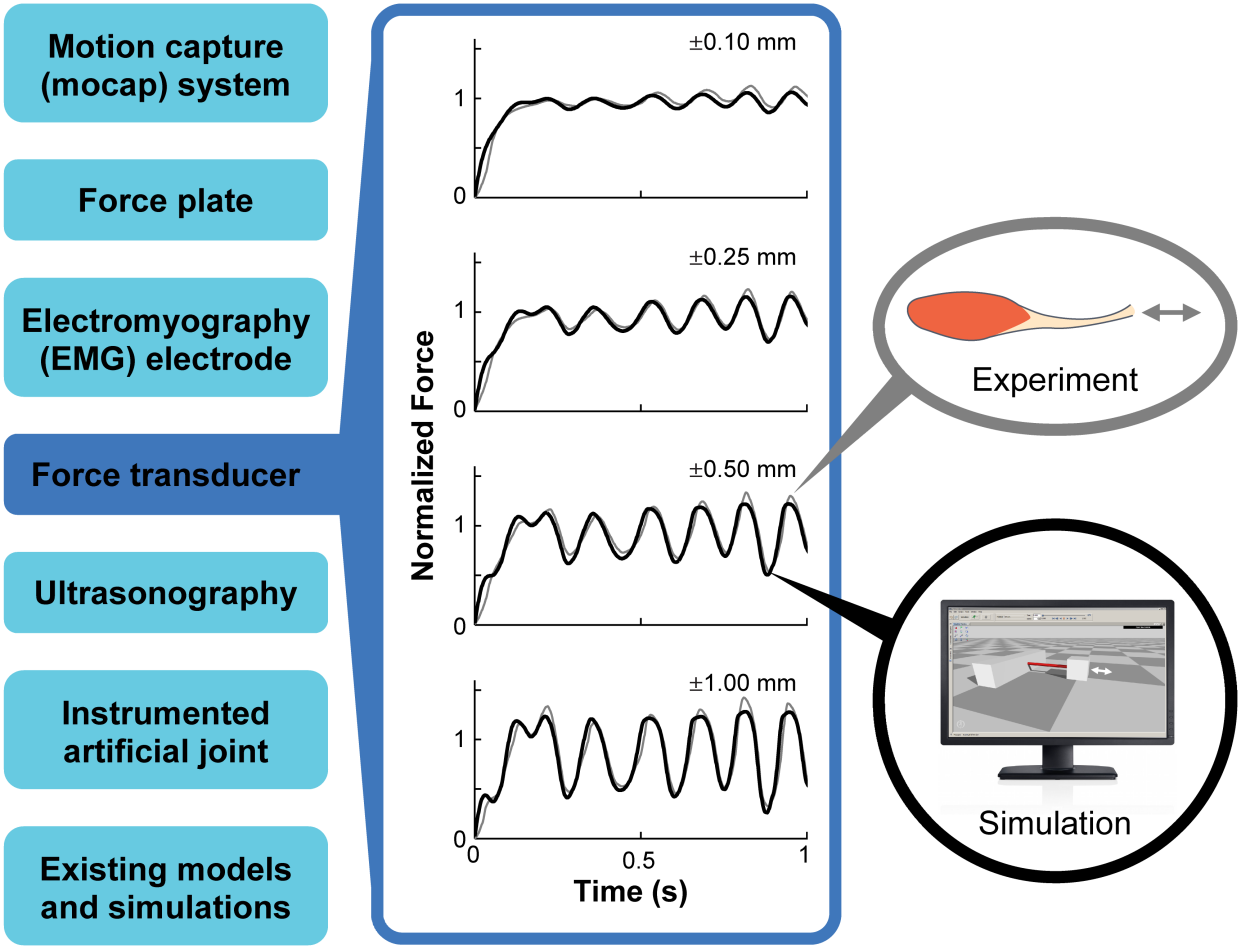
A variety of experimental and simulated data are used to validate OpenSim models. For example, our models of muscle contraction dynamics [46] were validated using *in vivo* isolated rat soleus muscle data from Krylow and Sandercock [51]. The data shown here (second column) were collected from one of these sources (force transducer; first column) as the muscle was maximally excited and its free end was displaced according to a predetermined time-varying signal, repeating for various displacements (shown here for 0.10–1.00 mm). We replicated these experiments in simulation to validate our computational model of muscle contraction dynamics [46].

OpenSim includes computational models of muscle, biological joints, and other musculoskeletal structures that are based on decades of research. Muscle mechanics, muscle architecture, and joint kinematics can be defined and modified to represent a wide variety of human and animal musculoskeletal structures. These capabilities are useful for modeling muscle spasticity and contracture in children with cerebral palsy [40], the unique musculoskeletal structures of animals to understand how ostriches run at high speed and with high metabolic economy [12], and in many other computational studies. Models of muscle mechanics have typically been validated against experimental data obtained from animals (e.g., the soleus muscle of rats; see Fig 5). These models have been adapted for studying human movement and can likewise be adapted to study a diversity of other animals. We have used recent experimental studies to improve previously published models and continue to add new models to expand the possible research applications of OpenSim. For example, biomechanical joints [38] compute accurate joint kinematics and reaction forces for the lower extremity (e.g., the knee [41, 42]), the spine and neck [43], and the shoulder [44]. The muscle models in OpenSim capture activation dynamics, the force–length and force–velocity relationships, and muscle–tendon dynamics [45]. Recent enhancements improve the computational speed and numerical stability of the muscle models as well as their agreement with *in vitro* testing of rat muscles [46]. Muscle metabolics models [13, 47–49] in OpenSim allow users to estimate muscle-level and whole-body energetics during movement. Neurophysiological structures and controllers, such as goal-directed and tracking (Computed Muscle Control; CMC) controllers [50] and a reflex controller [20], enable users to generate muscle-driven simulations of observed motions and to predict human and animal movement.

To ensure our models and simulations are biologically accurate, we validate our simulation results by comparing with experimental measures and other independent models and simulations (Fig 5). We also make all of our simulation and experimental data freely available so others can perform independent testing. Members of the OpenSim community are helping to validate models and simulations by performing sensitivity studies [52], developing benchmarks for multi-body system analysis [53], and analyzing parameter uncertainty [54].

### Custom simulation studies

OpenSim uses a modular Component and Solver architecture. Users can create custom simulation studies that combine existing computational tools in new ways, and write new computational tools that extend the built-in capabilities of OpenSim.

**Fig 6:**
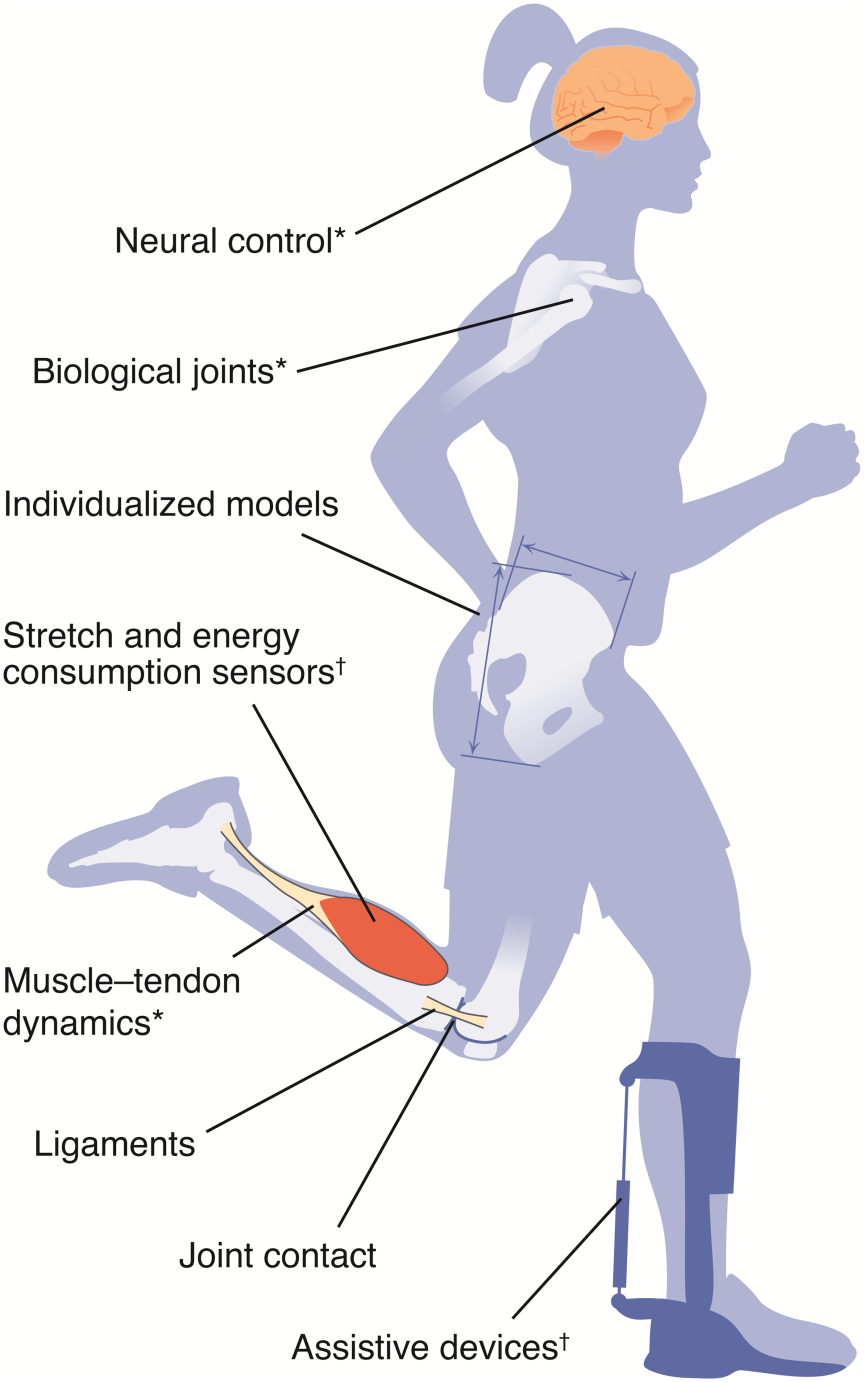
OpenSim enables physically accurate simulation of neuromusculoskeletal systems. Physics-based models of biological structures can be augmented with models of neuromotor controllers and mechatronic devices to reproduce and explain experimental observations, and to predict novel movements. OpenSim natively supports a wide variety of components, including those for modeling the skeleton as rigid bodies connected by joints, ligaments and other passive structures, muscles and motors, tracking and reflex-based controllers, external forces from the environment, and assistive devices composed of rigid bodies, joints, springs, and actuators. We have added new components to OpenSim (indicated with “†”) and enhanced many existing components (indicated with “*”). OpenSim’s collaborative, open-source development philosophy allows users to create, extend, and share new component models to accelerate their research.

OpenSim Components employ the composite design pattern [55], enabling users to assemble and combine models of musculoskeletal structures, exoskeletons, and implantable mechanisms to compute and report values of interest (Fig 6). A Component is a self-contained part of a Model that describes a physical structure or phenomenon and its contribution to the Model’s equations of motion. A variety of Components are built into OpenSim, including rigid bodies, joints, constraints, controllers, actuators, contact models, and springs. The computational models of muscle, biological joints, and other musculoskeletal structures described in the previous section are also Components. An OpenSim user builds a Model by specifying an interconnected set of Components. For example, a femur Body and a tibia Body are connected by a knee Joint, all of which are contained by the Model. This modular architecture allows users to systematically compose complex models from simpler submodels. The properties of the Component (e.g., the mass and inertia tensor of a Body) can be written to and read from a file (as XML), facilitating model archiving, editing, and sharing. Furthermore, users can write and share their own Components to extend OpenSim. For example, van der Krogt and colleagues created and validated a muscle spasticity controller and shared it as an OpenSim plugin on simtk.org [40].

OpenSim includes several inverse and forward Solvers to compute quantities of interest from a Model. For example, a forward dynamics Solver can be used to integrate model dynamics (i.e., state derivatives) forward in time to generate a trajectory of States. By incorporating reflex and other neurophysiological controllers, users can generate *de novo* movements. Alternately, inverse kinematics and dynamics Solvers determine the generalized coordinates (e.g., joint angles) and forces, respectively, that are consistent with external measurements (e.g., experimental marker trajectories from a motion capture system and ground reaction forces). OpenSim’s novel Component design provides access to the underlying dynamic equations, along with quantities like contact forces and metabolic cost, allowing users to extend OpenSim by creating custom Solvers. For example, single-shooting (e.g., [19–21]) and direct collocation (e.g., [56–60]) methods have been applied to predict movements like walking and jumping that optimize a user-determined objective (e.g., minimizing metabolic cost or maximizing jump height).

### Data flow

OpenSim provides users with flexibility and control when both inputting data, such as experimental measurements that drive a simulation, and outputting results of interest, such as joint angles and muscle forces. FileAdapters provide the capability to import data from common file formats (e.g., C3D, CSV, and TRC) and can be extended by developers to support new file types. Any Component can generate Outputs (e.g., a Muscle can output its force-generating capacity), which users can report to an internal table, a file, or the console using a Reporter. OpenSim also provides tools to manage the exchange of data within a simulation. Any Output generated by a Component (e.g., a Muscle’s fiber length) can be received by another Component as an Input (e.g., a stretch-based reflex Controller that generates an excitation signal based on a Muscle’s fiber length as an Input). DataTables are in-memory containers that can be used to store experimental and simulation data (e.g., marker locations, muscle-fiber lengths, and excitation signals) as columns of time series and their related metadata, such as column labels and units of measurement.

### Model visualization

Visualization of models and simulations is vital for interpreting, troubleshooting, and communicating results. OpenSim’s WebGL visualizer (see Fig 3) uses a modern graphics rendering pipeline, which provides features such as lighting, shadows, and textures, along with cross-platform support. Components can be visualized with analytic geometry (e.g., spheres, cylinders, and boxes; see the exoskeleton in Fig 3) that is straightforward to define, in addition to mesh-based geometry (e.g., bone meshes).

## Results

OpenSim is enabling researchers from diverse fields to gain insight into human and animal movement. Since its first release, OpenSim has been used as a modeling and visualization tool to examine the functional roles of individual muscles in human gait and to understand the effect of treatments on patients with gait disorders caused by cerebral palsy and stroke [2, 61–71]. Open access to human and animal models and recent improvements to the software (see Design and implementation) have expanded the scope of studies that are performed with OpenSim. Comparative biologists are using OpenSim to study relationships between form and function in animals [12, 72–76], and engineers are using OpenSim to design and analyze assistive devices [14–17]. Researchers are also using OpenSim to create models of reflexes and spinal circuits [20, 40, 77, 78] to understand movement disorders or prevent injury. OpenSim is being integrated into larger simulation and experimental frameworks for research in ergonomics [79], assistive robotics [80–82], and neurorehabilitation [78], where a model of human or animal movement is integral to the design of workspaces, devices, and treatments. The four examples below represent the range of studies enabled by recent advances in OpenSim.

### Example 1: Form and function

**Fig 7:**
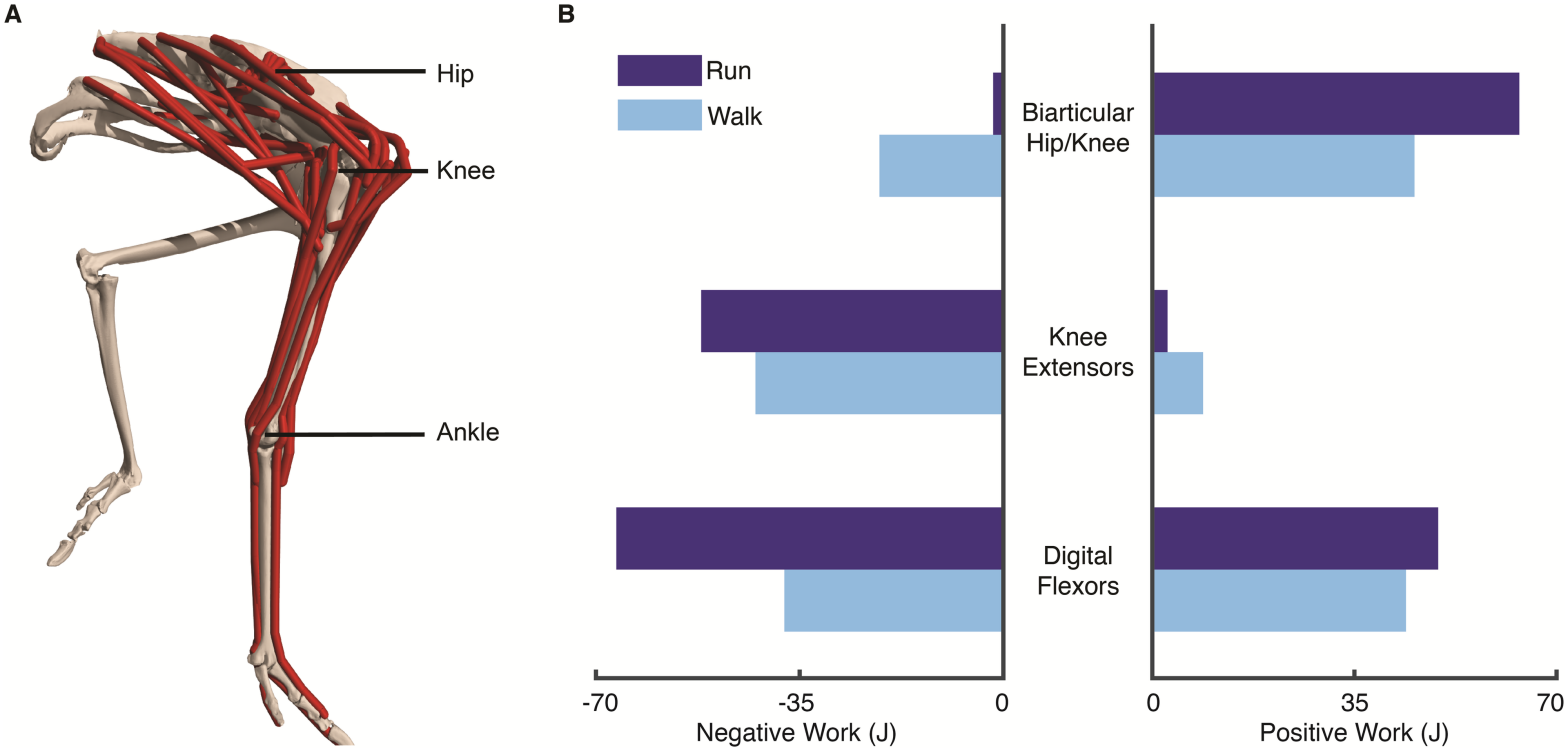
OpenSim facilitates defining anatomically accurate musculoskeletal models to reveal relationships between form and function. In this study, Rankin et al. [12] built a detailed model of an ostrich (*Struthio camelus*) pelvic limb in OpenSim (A) and collected motion capture data to generate simulations of ostrich locomotion. The researchers generated simulations of running (navy blue) and walking (light blue) with compliant tendons, using the Computed Muscle Control Tool in OpenSim. For each muscle group, they computed the negative and positive work performed by the muscles during stance (B) and swing (not shown). The biarticular muscles crossing both the hip and knee performed largely positive work during stance, contributing to propulsion, while the knee extensors performed negative work, acting as brakes. Adapted from Rankin et al. [12].

OpenSim is being used to discover relationships between form and function that explain how humans and animals move. For example, Rankin and colleagues [12] investigated the mechanisms responsible for the impressive speed, agility, and efficiency of the fastest running biped: the ostrich. The researchers constructed a musculoskeletal model of the ostrich’s lower limb (Fig 7A) and used an inverse approach in OpenSim to compute the muscle forces and mechanical work required to track experimental measurements of walking and running. Their analysis revealed that the biarticular muscles crossing both the hip and knee were the primary contributors to propulsion during stance, based on the large positive work they generate (Fig 7B, *biarticular hip/knee*). In contrast, the uniarticular knee extensors acted like brakes, performing negative work. The digital extensor muscles—which cross several joints, including the ankle and the metatarsal–phalangeal joints—and their compliant tendons acted like springs by storing energy (performing negative work) in early stance and releasing energy (performing positive work) in late stance, particularly during running (Fig 7B, *digital flexors*). The model, experimental data, and simulation procedures to reproduce their results are available on Dryad [83]. Analogous studies have been performed for other extant [35, 74, 75, 84] and extinct animals [10] to relate musculoskeletal form to function.

### Example 2: Device design

**Fig 8:**
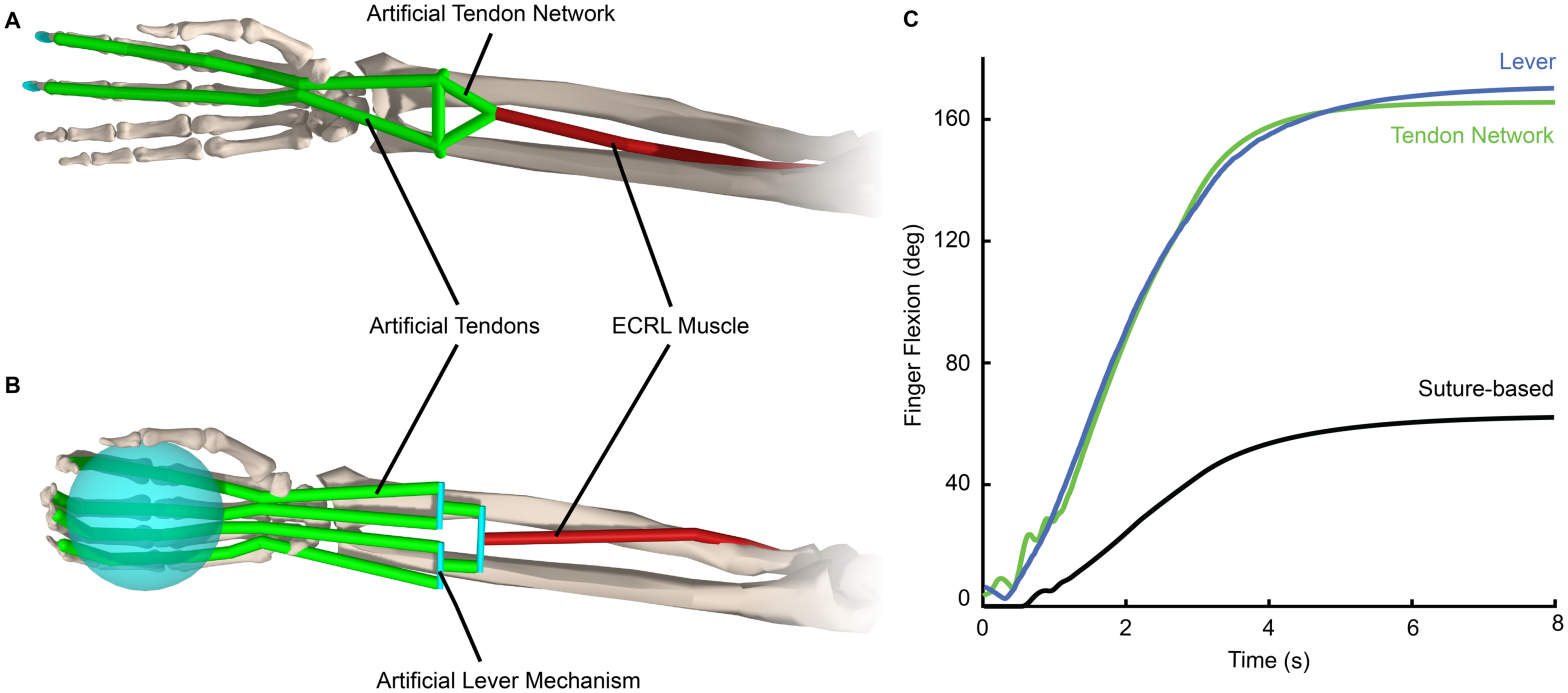
OpenSim supports design and analysis of implantable devices to restore grasp for those with paralysis. In current practice, individuals with partial paralysis of the upper limb receive tendon transfer surgeries to reconnect the tendons that facilitate finger movement to a non-paralyzed donor muscle. Homayouni and colleagues [14] are designing implantable devices to improve outcomes of traditional, suture-based tendon transfer. In one design (A), the single-suture attachment is replaced with an artificial tendon network. In a second design (B), a lever mechanism replaces the suture to more evenly distribute forces between the digits. The investigators used OpenSim to model the traditional suture-based surgery and each proposed design, and simulated a grasping motion. The implantable devices (green and blue) achieved greater finger flexion (C) than the traditional suture-based surgery (black). Adapted from Homayouni et al. [14].

OpenSim is being used to design implantable and exoskeletal devices. For example, Homayouni and colleagues [14] used OpenSim to prototype new passive, implantable mechanisms for hand tendon transfer surgery to improve grasp performance and restore function in patients with partial paralysis of the upper extremity. The goal of the mechanisms is to achieve a grasp that evenly distributes forces between the fingers—a key limitation of current tendon transfer procedures. The researchers modeled an artificial tendon network and a lever mechanism (Fig 8A and 8B), both of which act to distribute force from the attached extensor carpi radialis longus (ECRL) muscle among four tendons in the hand. They performed forward dynamics simulations of grasping tasks, where the motion was not prescribed but evolved based on the activation of the ECRL muscle. They examined the resulting kinematics of the digits of the hand and the forces applied to a grasped ball. Both proposed mechanisms allowed for greater motion of the digits (Fig 8C) while maintaining grasp strength comparable to that of healthy individuals. Others are using OpenSim to study assistive devices, such as those that assist walking and running [16–18], and to improve below-knee prosthetic comfort and performance [85].

### Example 3: Neural control

**Fig 9:**
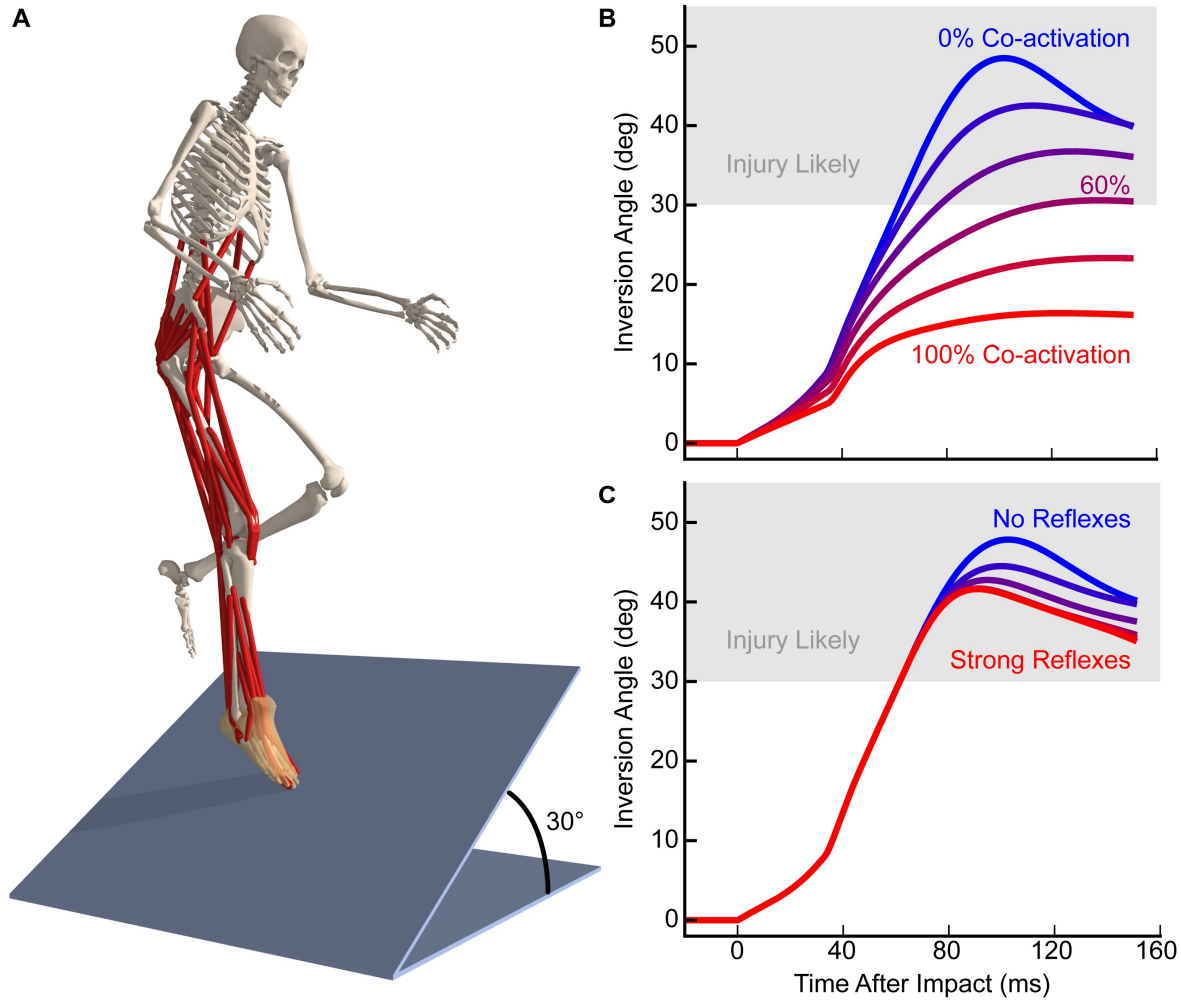
OpenSim reveals the roles of reflexes and preparatory co-activation in preventing ankle injury. DeMers and colleagues [20] created an OpenSim model to study risky landing scenarios (A), which included a detailed ankle joint to model both passive and active components, stretch-reflex controllers to actuate the muscles, and a contact model to estimate foot–floor reaction forces. They used the model to simulate a single-leg drop-landing onto an angled surface, which induced rapid ankle inversion. While preparatory co-activation of the invertor and evertor muscles (B) was able to prevent the ankle from inverting to angles that may cause injury (gray region), a reflex-only strategy (C) was not able to prevent injury in the scenario studied. Models and data are available on simtk.org [23]. Adapted from DeMers et al. [20].

The ability to create custom controllers in OpenSim enables users to investigate the role of reflexes in generating movement and preventing injury. Consider, for example, ankle sprains, which are the most common acute sport trauma [86]. Existing interventions have limited success [87, 88], in part because the role of muscle coordination in preventing injury is poorly understood [20]. DeMers and colleagues used OpenSim to compare the effectiveness of reflex control and preparatory co-activation in preventing ankle injuries. The authors simulated many landing scenarios, including those too dangerous to study experimentally. Their simulations used a full-body musculoskeletal model with muscle stretch reflexes and preset muscle activation controllers (Fig 9A). The model also included an elastic foundation contact model to compute foot–floor contact forces, and passive force elements that modeled the mechanics of ankle ligaments. Over a wide range of simulated landing scenarios, they found that strong preparatory co-activation of the ankle evertors and invertors prior to ground contact prevented the ankle inversion angle from exceeding injury thresholds (Fig 9B). Conversely, even superhuman stretch reflexes were too slow to generate adequate eversion moments before the simulations reached the threshold for inversion injury (Fig 9C). These results suggest that training interventions to protect the ankle should focus on stiffening the ankle with muscle co-activation prior to landing. The musculoskeletal and neuromuscular controller models, software, and simulation results from this study are freely available [23]. Previous studies using OpenSim have provided controller-driven simulations to examine the changes in muscle function due to factors like muscle spasticity [40] or surgical intervention [63–65, 77]. Further, by applying optimization to select controller parameters, researchers have been able to predict human-like movements [21] and adaptations to varying physiological and environmental conditions [19].

### Example 4: Multidisciplinary research

**Fig 10:**
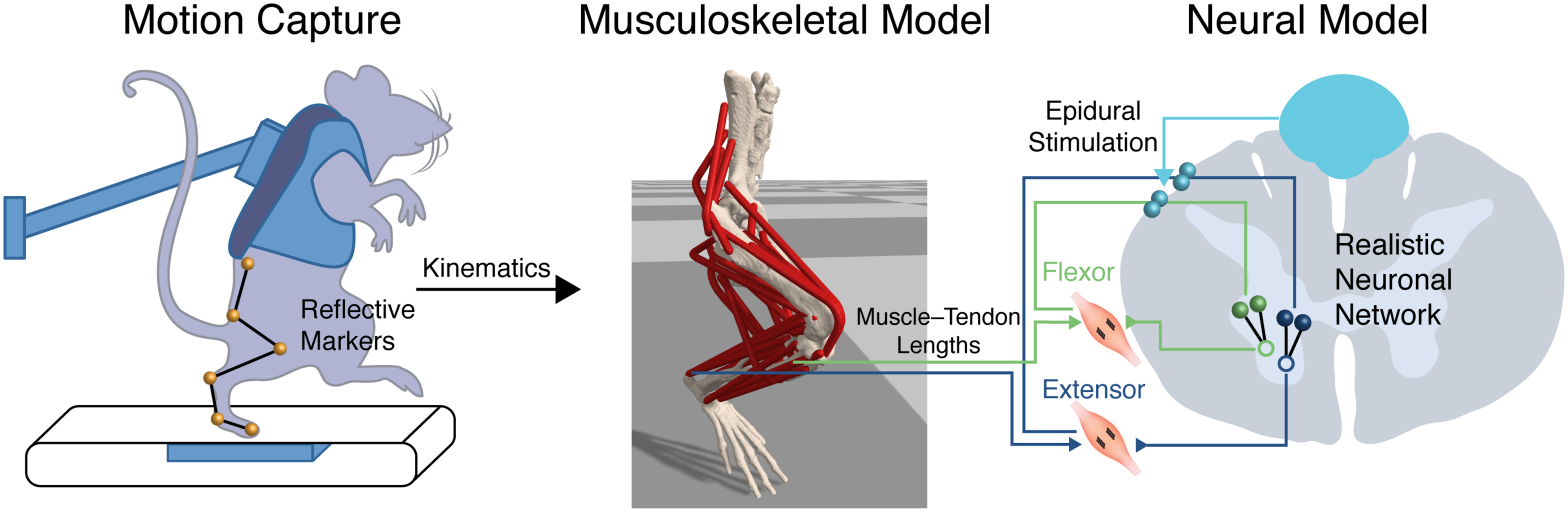
Combining neural and musculoskeletal models to study neuromodulation of spinal circuits for correcting motor deficits. Moraud et al. [78] measured the movement of spinal cord–injured rats (left panel; experimental setup with marker kinematics, ground reaction forces, and muscle electromyography (EMG)). A musculoskeletal model of the rat hindlimb (center panel) was developed in OpenSim to provide estimates of muscle fiber lengths and velocities from measured kinematics, which were inputs to muscle spindle models (right panel; black coils). Spindle reflexes from the major flexor and extensor muscles were the primary inputs to a realistic model of spinal neuronal circuits (right panel), which generated the neural drive to the same major muscle groups. The spindle reflexes were coupled to electrical epidural stimulation (EES) that modulated the spindle signals into the spinal circuits. The results (not shown) from Moraud et al. revealed that simulated neuromuscular activity successfully predicted changes of *in vivo* muscle activity (EMG) due to variations in EES frequency and amplitude. The OpenSim model of the rat hindlimb by Johnson et al. is available on simtk.org [90].

OpenSim enables investigators to incorporate musculoskeletal models into multidisciplinary simulation studies that require expertise and tools from diverse fields, such as neuroscience [78], ophthalmology [89], and human–machine interaction [80]. For example, electrical epidural stimulation has shown promise for restoring some voluntary function in individuals with spinal cord injury (SCI; Fig 10); however, the mechanisms by which the therapy enables modulation of muscle activity are poorly understood. Moraud et al. [78] used simulations to uncover potential mechanisms by which the stimulation can restore standing and walking, and inform the design of new stimulation patterns to improve function. OpenSim was integrated into a larger computational framework to provide biomechanically accurate estimates of muscle stretch in rats during locomotion—key inputs to the computational spindle circuit model of Moraud et al. The simulations revealed that epidural stimulation modulated muscle activity by interacting with muscle spindle feedback circuits such that increments in frequency ranging from 10 to 100 Hz led to a linear increase in the mean firing rate of both sensory and motor neurons. A comparable modulation in motor output was observed when increasing electrical epidural stimulation amplitude. These characterizations enabled the investigators to design phasic stimulation strategies for rats with incomplete and complete SCI, thereby reducing gait asymmetry and restoring balance in spinalized rats. Their findings and stimulation techniques could eventually help humans regain mobility after SCI.

## Availability and future directions

OpenSim is an open-source project with dozens of contributors and thousands of users. The OpenSim source code is available under the permissive Apache License 2.0, making OpenSim suitable for any academic, commercial, government, or personal use (some dependencies have more restrictive licenses). The source code is available on GitHub at https://github.com/opensim-org/opensim-core and https://github.com/opensim-org/opensim-gui. To make OpenSim accessible to a broad community of users, pre-packaged binaries with a desktop application (GUI and visualizer) are released periodically at https://simtk.org/projects/opensim. Documentation for users and developers, teaching materials, examples, musculoskeletal models, the Q&A Forum, and other resources can be accessed through http://opensim.stanford.edu/support/.

The recent software developments described above, coupled with the collective effort of the diverse and growing user community, will enable OpenSim to play an integral role in advancing research and design. Generating simulations of *de novo* movements (i.e., predictive simulation) is one area of particular interest. For example, providing easier access to predictive simulation would enable rehabilitation researchers to use simulations to guide the design of devices for preserving, restoring, and enhancing movement, and would accelerate the efforts of comparative biomechanists to predict the locomotor patterns of extinct species [91, 92]. Recent advances in direct collocation [57, 60, 93], fast and accurate impact models [94], and efficient modeling techniques [44] will help bring predictive simulation to a broader audience. As demonstrated by Moraud et al. [78], neural controllers can be studied experimentally by using a computational model of the musculoskeletal system as the plant in simulations of movement. The principles learned from such studies could eventually be used to improve control of implantable devices, prostheses, and exoskeletons (e.g., via real-time sensing and biofeedback). OpenSim can provide virtual prototyping capabilities for improving outcomes and reducing development time in clinical applications.

As with any open-source project, the future of OpenSim will be determined largely by the participation of the community. Our vision is to deepen the understanding of human and animal movement and to accelerate the development of rehabilitative treatments and assistive devices. Simulation can help realize this vision. Computer-aided design revolutionized the processes of conceptualizing, developing, and manufacturing engineered products from appliances to airplanes. We envision that neuromuscular and musculoskeletal simulation will continue to drive the evolution of engineered devices and treatments that assist and interact with people. The complexity and diversity of the human (and animal) motor system present fundamental challenges that can be overcome only by a large, diverse, and active community of researchers, engineers, and clinicians. This community benefits from models and simulation tools for making discoveries, transferring knowledge, and designing effective technologies. OpenSim is poised to support these endeavors.
